# Detection of viral antigen and inflammatory mediators in fatal pediatric dengue: a study on lung immunopathogenesis

**DOI:** 10.3389/fimmu.2025.1487284

**Published:** 2025-02-04

**Authors:** Leandro Junqueira Moragas, Laíza Vianna Arruda, Lucca de Lima Siqueira Oliveira, Felipe de Andrade Vieira Alves, Natália Gedeão Salomão, Jemima Fuentes Ribeiro da Silva, Carlos Alberto Basílio-de-Oliveira, Rodrigo Panno Basílio-de-Oliveira, Ronaldo Mohana-Borges, Caio Gonçalves Azevedo, Gabriela Xavier de Oliveira, Jorge José de Carvalho, Fernando Colonna Rosman, Marciano Viana Paes, Kíssila Rabelo

**Affiliations:** ^1^ Laboratório Interdisciplinar de Pesquisas Médicas, Instituto Oswaldo Cruz, Fundação Oswaldo Cruz, Rio de Janeiro, Brazil; ^2^ Laboratório de Ultraestrutura e Biologia Tecidual, Universidade do Estado do Rio de Janeiro, Rio de Janeiro, Brazil; ^3^ Laboratório das Interações Vírus-Hospedeiros, Instituto Oswaldo Cruz, Fundação Oswaldo Cruz, Rio de Janeiro, Brazil; ^4^ Departamento de Patologia, Faculdade de Medicina, Universidade Federal do Rio de Janeiro, Rio de Janeiro, Brazil; ^5^ Laboratório de Genômica Estrutural, Instituto de Biofísica Carlos Chagas Filho, Universidade Federal do Rio de Janeiro, Rio de Janeiro, Brazil; ^6^ Serviço de Anatomia Patológica, Hospital Municipal Jesus, Rio de Janeiro, Brazil

**Keywords:** dengue virus, children, histopathology, lung, inflammation

## Abstract

**Introduction:**

The dengue virus (DENV) is the etiological agent that causes dengue fever illness, an arbovirus with a major endemic potential that has become increasingly prevalent in Brazil and has already been associated with fatal cases in children. DENV has tropism for several organs, including lungs causing pulmonary complications. The aim of this article was to evaluate the inflammatory and histopathological profile of the lung tissue of three fatal cases of children infected with DENV, which represents a group more susceptible to fatality due to its incomplete development.

**Methods:**

Histopathological analysis was carried out using Hematoxylin and Eosin staining and special stains. While the characterization of the inflammatory response and cellular expression was done by marking the viral protein, macrophages, lymphocytes and pro-inflammatory cytokines.

**Results and discussion:**

The results confirm that vascular dysfunctions such as hemorrhage, vascular congestion and edema associated with a mononuclear infiltrate were observed in all three cases. In addition, the presence of viral replication and increased expression of inflammatory markers were also observed. Such findings contribute to the study and description of dengue, especially its effects on lung tissue.

## Graphical Abstract

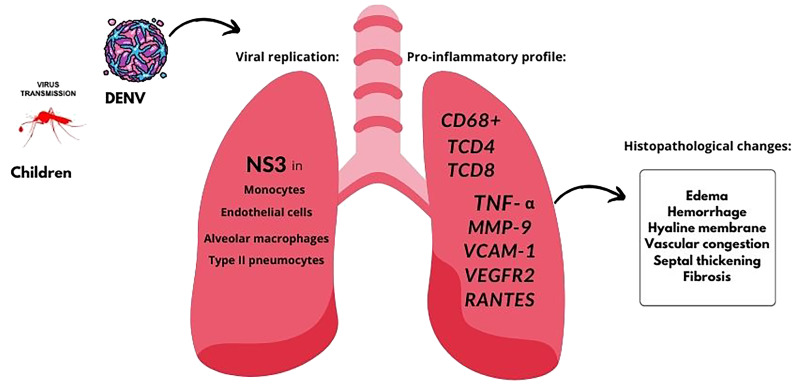

## 1 Introduction

Dengue fever (DF) is an arboviral disease that represents a global public health concern, especially in tropical and subtropical regions. Dengue virus (DENV) belongs to the *Flaviviridae* family transmitted mainly by mosquitoes of the *Aedes* genus (1). DF is endemic in several countries in Africa, Southeast Asia, the Western Pacific, and America (2, 3) and, during the last year, more than 5 million cases were reported globally (4). Brazil is one of the most affected countries, with a significant increase in the incidence of DF, reporting the highest number of cases (over 2.9 million) in America during the year of 2023 (4, 5). By June 2024, more than 6 million cases (6,121,980 cases) and around 4 thousand deaths from dengue fever had been registered in Brazil, indicating an increase in cases that constitutes a hyperendemic and emphasizes the importance of studying this disease in the country (6).

DENV comprises four distinct serotypes (DENV-1, DENV-2, DENV-3 and DENV-4), which are genetically related (7) and present similar clinical results – except in cases of secondary infection with a different serotype (8, 9). DENV infection commonly consists of a mild form, including symptoms such as arthralgia, headache, vomiting, myalgia, and skin rash (10). However, DF can progress to severe forms including dengue hemorrhagic fever (DHF) and dengue shock syndrome (DSS) (11). Some studies have pointed out that the most serious conditions of DENV infection present hemorrhagic symptoms, severe plasma leakage and severe involvement of multiple organs (12–14), which is usually present in children aged less than 15 years (15, 16). Therefore, mortality is directly associated with the complications of the disease, which leads to the impairment of several organs (7, 17).

Multiple organs can be affected by DENV infection, and several studies have already identified impairment of the central nervous system, skeletal muscle, liver, heart, and lungs (18–26). Furthermore, histopathological changes have been also observed in the spleen, kidney, pancreas and placenta (22, 24, 27–30). Lung involvement during DENV infection has been documented in several studies and is generally correlated with severe forms of the disease (26, 31–34). Thus, extensive lung involvement, especially associated with pulmonary hemorrhage, is a terminal event and is closely associated with uncontrolled infection and DSS (34).

Due to their immature hemodynamic system, children and specifically infants tend to develop severe dengue fever (35, 36) – with vascular leakage and shock being more frequent and severe in children than in adults (37, 38). Systemic vascular leak syndrome typically manifests itself at the time of defervescence and is characterized by progressive leukopenia associated with thrombocytopenia, hemorrhagic manifestations, pleural effusion, and ascites (36). To detect a serious change in the disease, the identification of different plasma extravasation markers, such as RANTES, VEGFR-2 and VCAM-1, is often used (27, 39, 40). This is important, because a few years ago there was an increase in the number of serious cases in the younger age group, highlighting the importance of investigating the pathophysiology of dengue, especially in this group (16, 36).

In this context, the present work aimed to investigate the histopathological aspects of lung tissues from three fatal cases of dengue in children that occurred between 2008 and 2012, as well as covering the inflammatory immune profile. It is known that the combination of inflammatory markers and vascular permeability in the febrile phase of dengue is associated with the development of more severe outcomes (40). Therefore, we focused on investigating the presence of immune cells with a chronic profile, pro-inflammatory cytokines such as TNF-a and vascular permeability markers in lung. These patients developed severe dengue fever, which critically affected other organs, besides the lungs, including the liver, as previously described by our group (41). Therefore, histopathological, and immunohistochemical analysis of lung tissue damage can help to elucidate the immunological mechanisms involved in the pathogenesis of dengue in the lung and how this contributed to the outcome of these patients.

## 2 Methods

### 2.1 Ethical considerations

All procedures performed during this study were approved by the Ethics Committee of the Oswaldo Cruz Foundation/FIOCRUZ (CAEE: 47525115.3.0000.5248).

### 2.2 Clinical history of fatal cases and controls

Lung tissue samples came from three children who died from DENV infection, during the outbreak that occurred in Rio de Janeiro, between 2008 and 2012. The diagnosis of dengue fever was confirmed in all patients, due to the presence of anti-DENV IgM antibodies or detection of NS1 antigen by immunochromatography. The clinical history of all patients and the respective conditions of each case are described in Moragas, L. J. et al., 2023 (41). The negative controls of lung samples were obtained from three fatalities ranging from 8 to 13 years old presenting no signs of infectious diseases or lung disorder.

### 2.3 Histopathological analysis

The lung tissue fragments from necropsies were fixed in 10% formaldehyde, pH 7.2, processed and blocked-in paraffin resin. 5 µm thick sections were made using a microtome (Leica, Germany) and mounted on glass slides. Before staining, slides were deparaffinized in three xylene baths and rehydrated with decreasing concentrations of ethanol (100 to 70%) and water. Then, the sections were subjected to standard and special staining with Hematoxylin and Eosin (H.E.), Periodic Acid-Schiff (PAS), PicroSirius Red and Masson’s trichrome, prepared for viewing under a light microscope (Olympus, Tokyo, Japan). Photomicrographs were captured using Image-Pro Plus version 7 software (Media Cybernetics).

### 2.4 Immunohistochemistry procedures

For detection of NS3 protein as well as characterization of cell populations, cytokines and inflammatory mediators by immunohistochemistry, sections were treated as described in a previous work (33). Sections were then incubated overnight at 4˚C with the following primary antibodies: anti-NS3 (produced in house, expressed in *Escherichia coli*, purified and inoculated in BALB/c mice; dilution 1:100), rabbit anti-human CD4 monoclonal antibody clone SP35 (Spring Bioscience, CA, USA; dilution 1:100), mouse anti-human CD8 monoclonal antibody clone C8/144B (Dako, CA, USA; dilution 1:200), mouse anti-human CD68 monoclonal antibody clone EBM11 (Dako, CA, USA; dilution 1:200), rabbit anti-human TNF-α polyclonal antibody clone ab6671 (Abcam, MA, USA; dilution 1:200), rabbit anti-human RANTES monoclonal antibody clone ab189841 (Abcam, MA, USA; dilution 1:200), rabbit anti-human VEGFR-2 monoclonal antibody clone SP123 (Spring Bioscience, CA, USA; dilution 1:100), rabbit anti-human VCAM-1 monoclonal antibody clone ab134047 (Abcam, MA, USA; dilution 1:100), mouse anti-human MMP-9 monoclonal antibody clone sc-21733 (Santa Cruz Biotechnology, TX, USA; dilution 1:100). On the second day, sections were washed three times and incubated with secondary antibody (REVEAL complement, Spring Bioscience, CA, USA) for 10 min and with rabbit anti-mouse IgG-HRP conjugate (REVEAL polyvalent HRP, Spring Bioscience, CA, USA) for 15 min at room temperature; followed by reveal of the reaction with the substrate for peroxidase diaminobenzidine (Dako, CA, USA). Counterstaining was performed with Harry’s hematoxylin (Sigma, MO, USA) and then, sections were prepared for visualization under a light microscope (Olympus, Tokyo, Japan).

### 2.5 Damage quantification and statistical analysis

Quantification of lung lesions were performed by a semi quantitative analysis in sections stained with H.E. For each of the parameters used in the quantification of damages (septal thickening, hyaline membrane formation, degree of edema and hemorrhage, in addition to the presence of inflammatory infiltrate), a numerical scale ranging from 0 to 4 according to the severity and the extent of damage was assigned (0 = none, 1 = mild, 2 = moderate, 3 = severe and focally, 4 = severe and diffuse).The values ​​were measured covering 20 randomly acquired fields at 1000x magnification, visualized by light microscopy.

Data were analyzed with GraphPad Prism software version 8.0 (La Jolla, CA, USA) using non-parametric statistical tests. Significant statistical differences between the analyzed groups (controls and cases) were determined using Mann-Whitney test with a threshold of p < 0.05.

## 3 Results

### 3.1 Histopathological analysis

**Figure 1:**
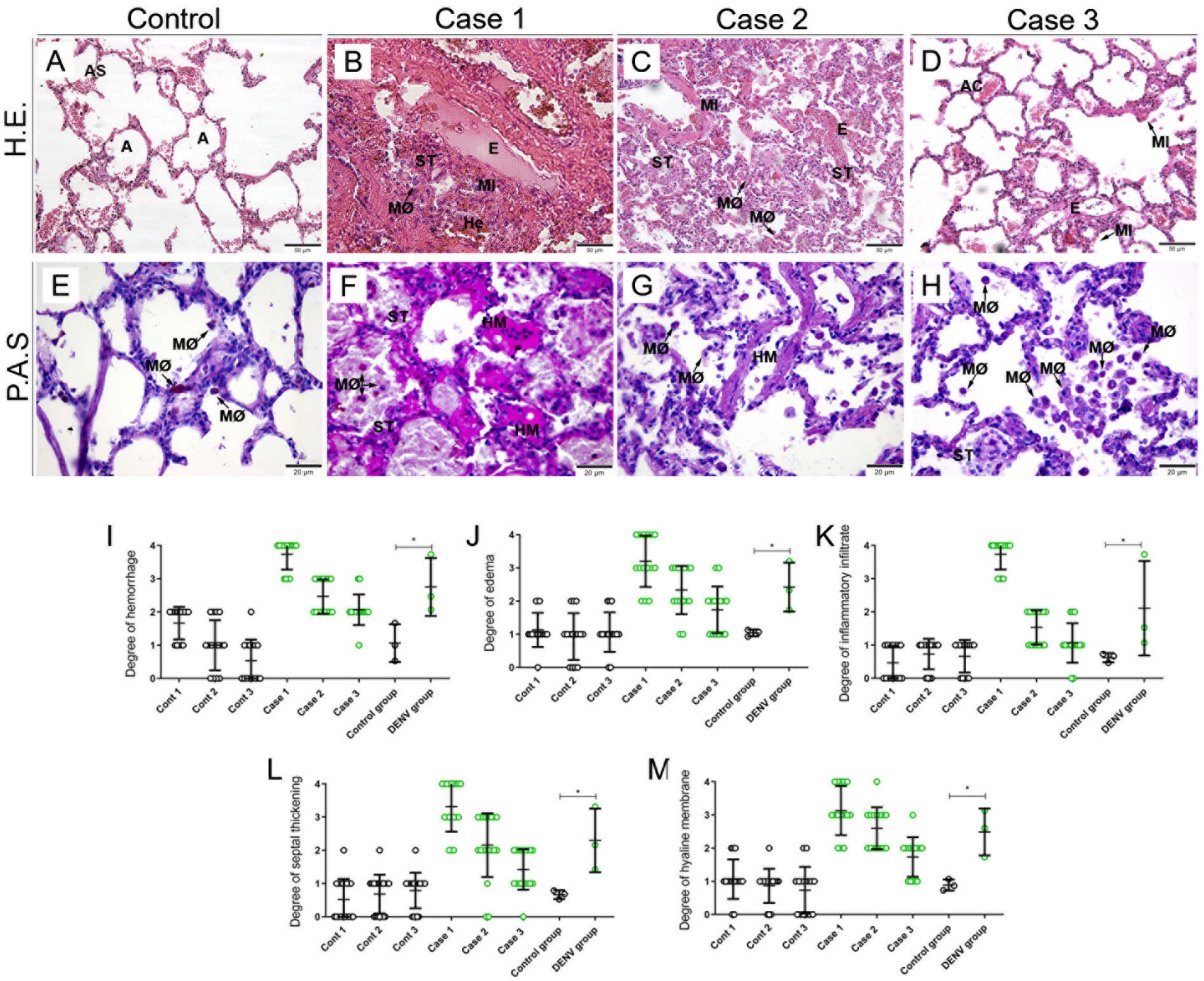
Histopathological aspects of lung tissues from fatal cases of DENV in children and respective controls. **(A, E)** Lung from non-dengue cases stained with H.E. and PAS, respectively showing preserved alveoli and normal appearance. Lung sections from fatal dengue cases showing alveolar macrophage hyperplasia **(B, C)**, as well as areas of hemorrhage **(B)**, septal thickening **(B, C)** and edema associated with inflammatory infiltrate **(B-D)**. **(F-H)**. Special PAS staining reveals glycogen deposits in alveolar septa associated with the formation of hyaline membrane, in addition to intense positive staining in alveolar macrophages. (AS) Alveolar Septum; **(A)** Alveolus; (MØ) Macrophage; (He) Hemorrhage; **(E)** Edema; (MI) Mononuclear Infiltrate; (ST) Alveolar Thickening; (AC) Alveolar Capillary; (HM) Hyaline Membrane. **(I-M)** Semiquantitative analysis of changes in the lung tissue of infected and control children. Asterisks indicate significant differences by the statistical test (*p < 0.05). **(A-D)** Magnification 20x, **(E-H)** magnification 40x.

The control tissue samples presented a regular structure, with no sign of rupture, edema, hemorrhage or other morphological disorder (**Figure 1A**). On the other hand, the three cases presented similar histopathological changes: areas of septal thickening and edema associated with a significant mononuclear inflammatory infiltrate (**Figures 1B-D**) which suggests vascular permeability alteration. Furthermore, it was possible to observe alveolar macrophage hyperplasia (**Figures 1B, C**). Also, the presence of positive PAS staining revealed the formation of hyaline membrane in areas of the alveolar septum (**Figures 1F-H**), compared to the control, which showed discrete PAS staining (**Figure 1E**). Intense staining in alveolar macrophages together with hyperplasia, suggests activation of these cells (**Figures 1F-H**).

Semi quantitative damage analysis was conducted using five distinct parameters: hemorrhage, edema, inflammatory infiltrate, septum thickening and hyaline membrane formation. Unlike the control, which showed no or low impairment, in all cases we observed septum thickening, formation of a hyaline membrane, presence of edema, hemorrhage and inflammatory infiltrate in different degrees (**Figures 1I-M**). Cases 2 and 3 presented a moderate degree in the five criteria used, while in case 1 we observed a high degree of inflammatory infiltrate and severe tissue involvement in relation to septum thickening, formation of hyaline membrane, presence of edema and hemorrhage (**Figure 1I-M**).

### 3.2 Evaluation of fibrosis by collagen deposition and matrix metalloproteinase-9 expression and in lung tissues

**Figure 2:**
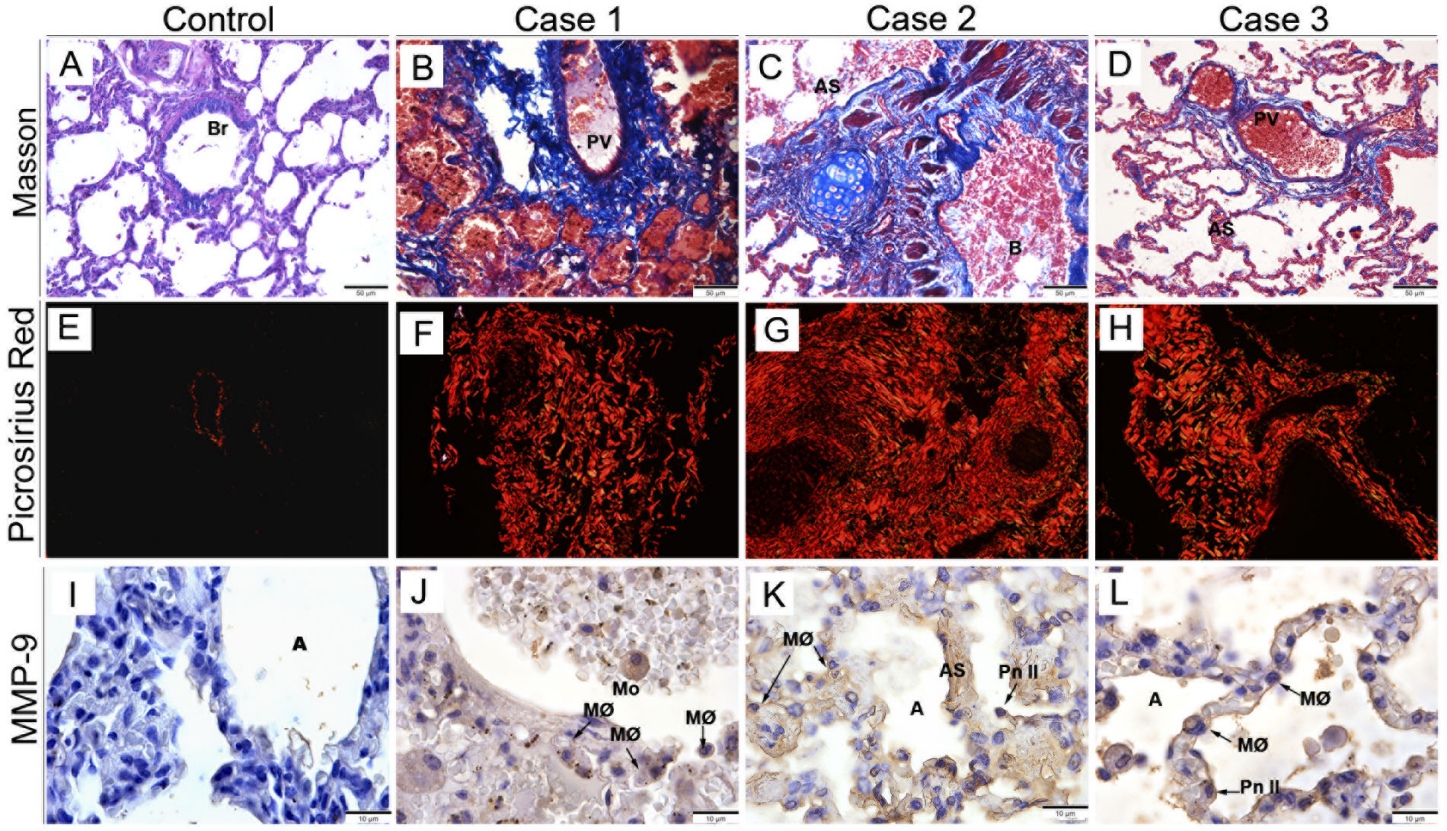
Analysis of collagen deposition in lung tissue and MMP-9 expression. Control case, stained with Masson’s trichrome **(A)** and Picrosirius red **(E)**, showing a regular distribution of collagen fibers around the bronchiole and pulmonary vein. Infected lung tissue stained with Masson’s trichrome, demonstrating deposition of collagen fibers disseminated throughout the alveolar septa **(B, C)**, around the pulmonary vein **(B, D)**, and bronchiole **(C)**. **(F-H)** Picro Sirius Red staining showing intense collagen deposition around the pulmonary vein and disseminated throughout the parenchyma. **(I)** Control lung tissues without MMP-9 expression. Detection of MMP-9 in alveolar macrophages **(J-L)**, type II pneumocytes, and inside alveolar septa **(K)**. **(B)** Bronchus; (Br) Bronchiole; (Pv) Pulmonary vein; **(A)** Alveolus; (MØ) Macrophage; (Mo) Monocyte; (AS) Alveolar Septum; (Pn II) Type II Pneumocyte. **(A-D)** Magnification 20x, **(E-H)** magnification 40x, **(I-L)** magnification 10x.

To evaluate the presence of fibrosis, we analyzed the deposition of collagen in areas of the lung by staining with Masson’s Trichrome and Picro Sirius Red. Furthermore, we detected metalloproteinase-9 (MMP-9), an enzyme that degrades type IV collagen and is a key modulator of the extracellular matrix and basement membrane. As expected, the control samples showed regular collagen deposition with an absence of MMP9 labeling (**Figures 2A, E, I**). In infected tissue, staining with Masson’s trichrome and Picro Sirius Red showed an increase in the deposition of collagen fibers around the pulmonary vein and, in regions of the parenchyma of some cases - coinciding with the alveolar septa and bronchi, suggesting areas of pulmonary fibrosis (**Figures 2B, C, D, F-H**). Furthermore, we detected the presence of MMP-9 in thickened alveolar septa (**Figures 2J-L**) inside alveolar macrophages, and type II pneumocytes.

### 3.3 Detection of viral antigen

**Figure 3:**
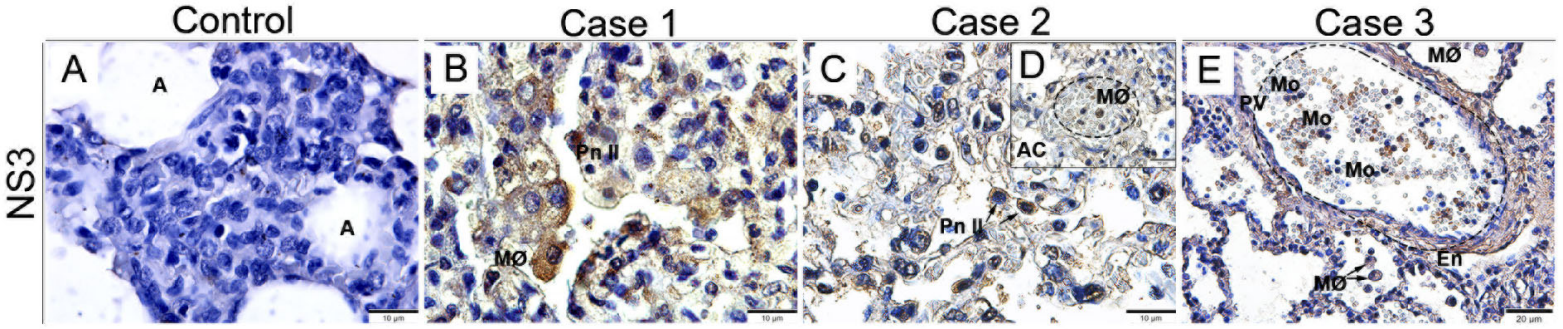
Detection of the NS3 protein antigen in lung tissues from DENV fatal cases of children. **(A)** Control lung tissue showing no presence of NS3 protein antigen. Detection of NS3 antigen in type II pneumocytes **(B, C)**, macrophages inside the alveolar capillaries **(D)**, and monocytes inside the pulmonary vein **(E)**. **(A)** Alveolus; (MØ) Macrophages; (Pn II) Type II Pneumocyte; (AC) Alveolar Capillary; (Mo) Monocyte; (PV) Pulmonary vein. **(A-D)** Magnification 100x, **(E)** magnification 40.

To investigate sites of viral replication in the lung tissues of the studied cases, it was performed the detection of the dengue nonstructural 3 (NS3) protein by immunohistochemistry assay. Controls (non-dengue cases) did not react with antibodies targeting NS3 antigen, revealing absence of viral replication in these tissues (**Figure 3A**). Positive reaction for NS3 antigen was indeed observed in all three dengue cases, mainly in type II pneumocytes (**Figures 3B-E**) and alveolar macrophages (**Figures 3B-D**), evidencing viral replication in these cells. The viral antigen was also detected in alveolar capillaries.

### 3.4 Analysis of the lymphocyte profile in infected lung tissues

**Figure 4:**
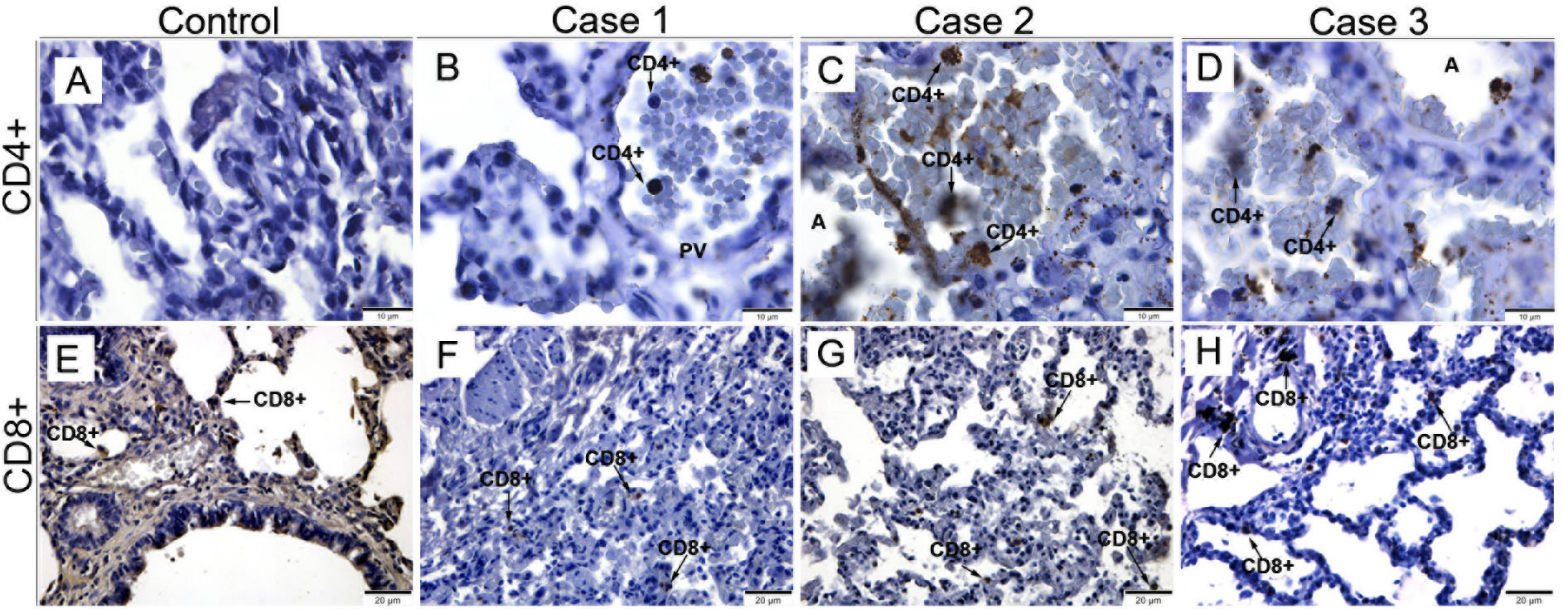
Profile of T lymphocytes in lung tissues from fatal cases of DENV in children. **(A, E)** Control cases with reduced expression of CD4^+^ and CD8^+^ T cells. Infected lung tissues, showing CD4^+^ T lymphocytes inside the pulmonary vein **(B)** and in the alveoli **(C, D)**. **(F-H)** CD8^+^ cytotoxic T lymphocytes detected in areas of septal thickening. (CD4^+^) CD4^+^ T Lymphocytes; (PV) Pulmonary vein; (CD8^+^) CD8^+^ T Lymphocytes. **(A-D)** Magnification 100x, **(E-H)** magnification 40x.

Areas with infiltrate observed in the lung tissue were characterized by the expression of CD4^+^ and CD8^+^ T cells. In both control samples, staining was observed as expected: few or none CD4^+^ T cells and CD8^+^ T cells homogeneously dispersed in the lung parenchyma (**Figures 4A, E**). In general, in all three cases we observe the presence of CD4^+^ T cells inside the pulmonary vein (**Figure 4B**) and associated with areas of hemorrhage in the alveoli (**Figures 4C, D**). Furthermore, we observed an increase in the number of CD8^+^ T cells, cytotoxic T lymphocytes, in the regions of septal thickening and spread throughout the parenchyma (**Figures 4F-H**) compared to the control.

### 3.5 Detection of macrophages and pro-inflammatory cytokine

**Figure 5:**
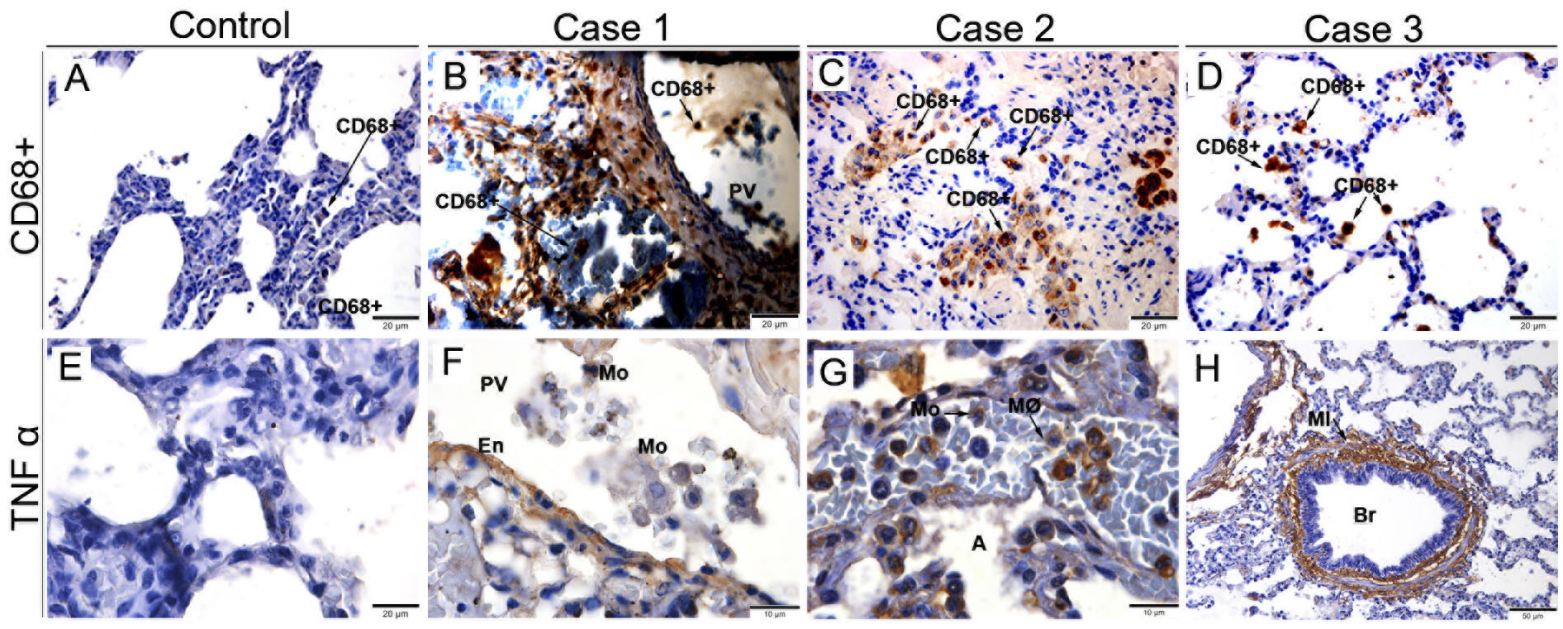
Characterization of macrophages and expression of TNF-α in fatal cases of DENV in children. **(A)** Control lung tissue with little expression of CD68^+^ macrophages. **(B-D)** Infected tissues expressing CD68+ macrophages throughout the lung parenchyma. **(E)** Control tissue presenting no expression of TNF-α. Expression of TNF-α in pulmonary vein endothelial cells **(F)**, in Monocytes **(G)**, and in the peribronchiolar Mononuclear infiltrate **(H)**. (CD68+) CD68+ Macrophages; (PV) Pulmonary vein; (En) Endothelium; (Mo) Monocyte; (MØ) Macrophages; (MI) Mononuclear infiltrate; (Br) Bronchiole. **(A-E)** Magnification 40x, **(F, G)** magnification 100X, **(H)** magnification 20x.

To identify the profile of macrophages and cytokines present in the tissue of infected cases, we detected CD68^+^ cells and the pro-inflammatory cytokine TNF-α. In the control tissue, we observed the expected labeling of CD68^+^ cells in discrete areas in the lung alveoli and the absence of TNF-α labeling (**Figures 5A, E**). In infected tissues, we observed positive staining of CD68^+^ cells in regions of septal thickening, around the pulmonary vein (**Figure 5B**), in scattered throughout the lung parenchyma (**Figure 5C**) and in focal areas of the alveoli (**Figure 5D**). In addition, we detected the expression of TNF-α in the endothelial cells around the pulmonary vein (**Figure 5F**), inside monocytes and alveolar macrophages (**Figure 5G**), as well as in the peribronchiolar mononuclear infiltrate (**Figure 5H**).

### 3.6 Analysis of mediators involved in vascular permeability in lung tissues

**Figure 6:**
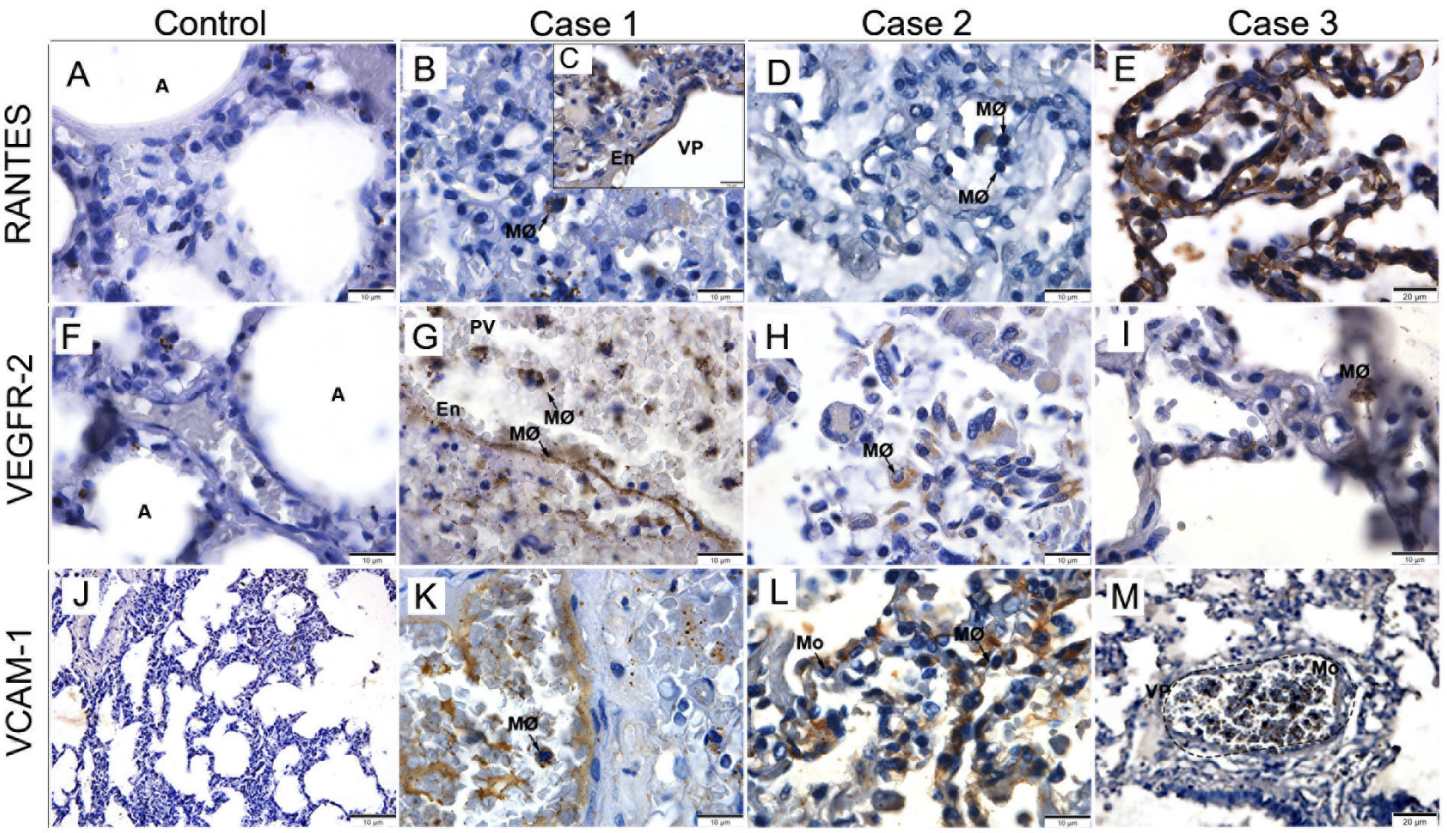
Characterization of vascular permeability in lung tissues from fatal cases of DENV in children. **(A, F, J)** Control tissues presenting little or no expression of RANTES, VEGFR-2 and VCAM-1. **(B-E)** Expression of RANTES in macrophages and endothelial cells of the pulmonary vein. **(G-I)** VEGFR-2 expressed in alveolar macrophages. **(K-M)** VCAM-1 expressed in circulating macrophages and monocytes. **(A)** Alveolus; (MØ) Macrophages; (PV) Pulmonary vein; (En) Endothelium; (Mo) Monocyte. **(A-D, F-L)** Magnification 100x, **(E, M)** magnification 40x.

The profile of mediators involved in vascular permeability and pro-inflammatory response was characterized mainly by the expression of RANTES, VEGFR-2 and VCAM-1, well-known markers implicated in the pathogenesis and progression of DENV disease. There was little or no detection of any of the mediators in control tissues (**Figures 6A, F, J**). On the other hand, in the patients’ tissues, we detected positive staining for RANTES inside macrophages, in the endothelial cells of the pulmonary veins (**Figures 6B-E**). Similarly, VEGFR-2 was detected in alveolar monocytes and macrophages and in pulmonary vein endothelial cells and alveolar capillaries (**Figures 6G-I**). Furthermore, we observed positive staining for VCAM-1 in circulating macrophages and monocytes, as well as in pulmonary vein endothelial cells (**Figures 6K-M**).

## 4 Discussion

In studies involving human cases of dengue, the involvement of various organs during the course of the disease has been increasingly observed, especially in fatal cases (29, 42–52). However, few studies have targeted children, who have recently shown a high rate of conversion from classical dengue to severe dengue. Therefore, this study focused on this age group.

Regarding lung findings, we observed that areas of hemorrhage, edema, septal thickening and hyaline membrane formation are characteristics of dengue shock, which adds to the severity of fatal outcomes. Previous studies conducted in our group had similar results (29, 49). Additionally, in the present study, we observed hyperplasia of alveolar macrophages and areas with mononuclear infiltrate. Increase in cellularity, presence of mononuclear infiltrate and hyperplasia of alveolar macrophages have been previously reported in adult patients as complications resulting from DENV infection (29, 49).

The NS3 viral antigen was detected inside type II pneumocytes, alveolar macrophages and within the alveolar capillaries. These findings demonstrate that DENV was able to infect these cells and replicate, once NS3 protein participates actively in viral replication. Similar results were found by our group in previous studies with the lungs of adult patients, where NS3 antigen was detected inside alveolar macrophages, type II pneumocytes and endothelial cells (49). NS3 is a common target for observing viral replication, since it is a protease that remains only in the cytosol. It has been studied as a therapeutic target to prevent viral replication and disease progression (50, 51).

Masson’s Trichrome and Picro Sirius Red staining were used for better evidence of collagen fiber and we observed an increase in the expression of these fibers, particularly around the pulmonary vein. We also noted increased expression of these fibers disseminated throughout the parenchyma in some cases, coinciding with the alveolar septa and some bronchi. Previous studies have also observed an increase in collagen fiber expression in the region of the alveolar septa, promoting septal thickening (53). Through PAS staining, we observed positive marking, indicating a high concentration of carbohydrates (glycoproteins), which are normally found in connective tissues, mucus and the basement membrane. It was more evident in the alveolar septa, indicating the presence of a hyaline membrane. High concentrations of hyaline membrane were already reported in patients with severe dengue, suggesting DENV shock (29, 49, 54).

We also identify a greater presence of inflammatory cells (CD8^+^ T and CD4^+^ lymphocytes and CD68^+^ macrophages), mainly in regions of septal thickening, diffuse in the parenchyma and around the pulmonary vein. It is known that increased presence of CD4^+^ and CD8^+^ T lymphocytes in the lung is associated with Chronic Obstructive Pulmonary Disease (COPD), as it is believed that CD8^+^ T lymphocytes cause modifications in the elastic fibers and musculature of the bronchi. The bronchioles can become obstructed by fibrosis and infiltration of macrophages (CD68^+^) and CD8^+^ T lymphocytes. It is believed that macrophages and CD8^+^ T lymphocytes have important role in apoptosis and destruction of epithelial cells in the alveolar wall, releasing TNF-α, perforins and granzymes, in addition to stimulating the production of collagen fibers which induces fibrosis (55).

TNF-α expression was observed in the endothelial cells of the pulmonary vein, in monocytes and alveolar macrophages. Additionally, there was significant marking in a peribronchiolar mononuclear infiltrate. It is known that TNF-α is a cytokine involved in various inflammatory and fibrotic dysfunctions, playing an important role in the impairment of lung function. Anti-TNF-α therapies have shown good effects in controlling chronic pulmonary inflammatory response (56). As one of the main cytokines in the cytokine storm, TNF-a is a marker of disease severity and may be associated with severe infection. Studies with large numbers of patients confirm this association with outcomes in shock syndrome and dengue hemorrhagic fever (57). In addition, TNF-a was observes as one of the most prominent cytokines in the evaluation of immunoclusters in severe dengue in children (58).

There was an increase in the expression of other inflammatory mediators related to increased vascular permeability and cause of shock and deaths in dengue: RANTES, VEGF-R2, and VCAM-1. In our study, we observed higher expression of RANTES inside macrophages and endothelial cells of the pulmonary vein and pulmonary capillaries. RANTES has been described as responsible for the recruitment of T lymphocytes and blood monocytes, leading to an increase in alveolar macrophages and CD8^+^ T lymphocytes in the pulmonary parenchyma, contributing to the COPD condition (59). VEGFR-2 was detected in the endothelial cells of the pulmonary vein and alveolar capillaries, as well as in monocytes and alveolar macrophages. When bound to VEGF, it promotes the division, proliferation, and migration of endothelial cells, increasing vascular permeability and, consequently, plasma leakage (30, 60). Increased expressions of VCAM-1 were observed in macrophages, circulating monocytes, and endothelial cells of the pulmonary vein. This cytokine promotes the adhesion of lymphocytes, monocytes, eosinophils, and basophils to the vascular endothelium. Studies have found high concentrations of VCAM-1 in fibroblasts, collagen synthesizers (61), which may favor the formation of the hyaline membrane.

This study is one of the few focused on the immunopathogenesis of dengue in children. The most worrying aspect is that dengue cases in children tend to progress to severe dengue. It is not known for certain why children are more susceptible to severe dengue. We believe it may be due to the immaturity of the immune system and an inability to fight DENV, in which the side effects of cytokines and inflammatory mediators are more damaging than the disease itself. The increased presence of CD4^+^ T cells, CD8^+^ T cells and CD68^+^ macrophages indicate an exaggerated inflammatory response, which can culminate in dengue shock syndrome through the triggering of cytokines and inflammatory mediators. Investigating the main cells and inflammatory mediators involved in the pathogenesis of dengue contributes to clarifying the main mechanisms involved during an infection. In this way, we will be able to modulate the immune response, avoiding the deleterious effects of its activation and thus preventing progression to severe dengue and death. With this study, we have further clarified the immunopathogenesis of the disease, evaluating the possible biomarkers of severe dengue in order to identify them in the early stages of the disease and establish the best protocol for treatment.

## Data Availability

The original contributions presented in the study are included in the article/supplementary material. Further inquiries can be directed to the corresponding author.
